# Complexity of developing a nationwide model for a dementia registry in Egypt: A qualitative study

**DOI:** 10.1002/alz.70011

**Published:** 2025-02-26

**Authors:** Shimaa A. Heikal, Ghada Barsoum, Mohamed Salama

**Affiliations:** ^1^ Public Policy and Administration Department The American University in Cairo Cairo Egypt; ^2^ Institute of Global Health and Human Ecology The American University in Cairo Cairo Egypt

**Keywords:** Alzheimer's disease, dataset, dementia, Egypt, registry

## Abstract

**INTRODUCTION:**

The increased interest in evidence‐based medicine has led to the emergence of disease registries worldwide to help tackle the impact of chronic diseases. A national dementia registry in Egypt would provide a valuable source of patient data that will significantly advance the disease management strategies, quality of patient care, and impact health policy‐ and decision making.

**METHODS:**

This study investigates the complexity of creating a disease registry for dementia in Egypt by interviewing 24 experts to provide recommendations for the most appropriate registry model to be developed.

**RESULTS:**

Several core themes emerged from the analysis discussing many points that should direct the creation of any registry in Egypt in terms of functionality, planning, comprehension, governance, ethics, and challenges to avoid.

**DISCUSSION:**

Developing such a dataset is beneficial to the Egyptian health‐care system, which makes the investment worthwhile. Support and collaborative work from all stakeholders, along with suitable funding, are essential elements of the proper implementation of the project.

**Highlights:**

- Building a dementia registry is essential to progress research in understudied populations
- Qualitative analysis of 24 experts interviews provided insightful plan for dementia registry
- Core domains to address include: functionality, planning, comprehension, governance, ethics, and challenges to avoid.

## 1 BACKGROUND

The imperative to evaluate and improve the outcomes of disease management strategies in health‐care system settings has led to an increased interest in evidence‐based medicine. The need to promptly assess the interventions used for different groups of patients has increased the recognition of the role of observational studies and medical datasets.^1^ Conventional randomized clinical trials (RCTs) have shown critical gaps in evaluating the safety of the developed intervention and failed to rein in its effectiveness with an extensive array of the population over prolonged times.^2^ In addition, the lack of real‐world patient data hinders research in different areas, which hampers our knowledge of chronic and rare diseases, their risk factors, epidemiologic and economic burden, and, most importantly, the best practices to tackle them.^3^, ^4^ Moreover, the availability of accurate information on health outcomes is crucial for policy makers to support the development of health spending strategies to improve the value of health‐care services while decreasing costs.^5^

A systematic and comprehensive approach to caring for patients with chronic diseases has already shown great potential in improving the quality of patient care, which will lead to a decrease in disease costs that represent a global health burden in disease registries.^5^ Disease registries are effective tools that provide the required systemic and comprehensive data, which help health‐care providers integrate and use this information in their care settings.^6^, ^7^ A well‐established disease registry enables providers to track cases in their country and analyze their data to manipulate patients’' treatment effectively. In addition, registries are considered one of the ways to cost‐effectively collect longitudinal patient information to provide datasets that can be analyzed to give insights into different perspectives of the disease.^8^ The availability of accurate patient datasets, together with the big data analytics advancements, will tremendously impact the determination of disease causes and risk factors, resulting in the identification of improved practices for disease management and prevention.^9^

Age‐associated chronic diseases, including dementia and Alzheimer's disease (AD), are a vast and growing problem for human society.^10^, ^11^ The total number of people diagnosed with dementia is projected to increase from ≈ 50 million to 152 million people in 2050.^12^ Moreover, the total cost of dementia care was estimated at $818 billion in 2015, with lower costs per person in lower‐income countries; the cost per person was estimated as $32,865 in high‐income countries, $6827 in upper–middle‐income countries, $3109 in lower–middle‐income countries such as Egypt, and $868 in low‐income countries.^13^, ^14^ Yet, the epidemiological data regarding dementia and AD are mostly accurate when provided from high‐income countries, while most low‐ and middle‐income countries rarely have national data and rely on regional or international estimates.^15^, ^16^ Egypt has a national prevalence study that the World Health Organization recognized as a reference for the prevalence studies developed by the Middle East or North Africa region countries, but the study did not cover the entire country and only focused on a few cities in Upper Egypt.^15^, ^17^

**RESEARCH IN CONTEXT**

1. **Systematic review**: The authors reviewed the literature using traditional databases (e.g., Scopus, Embase, and PubMed), meeting abstracts, and published reports. While developing a disease registry is not yet widely studied in Egypt, several recent publications have investigated Dementia registries worldwide. These relevant citations are appropriately cited.
2. **Interpretation**: Our findings led to identifying the best registry model to develop for Egypt. This model is consistent with the best practices reported worldwide to implement such a database.
3. **Future directions**: The manuscript proposes a framework for the establishment of a national disease registry for Dementia. The evidence we got through the discussions formulated the key steps that need to be followed in order to develop this kind of registry in Egypt. Additional efforts and actions are required in terms of policy and legislation to support the success and functionality of the registry once established

The current study aims to generate a discussion to assess the feasibility of creating a disease registry in Egypt and garnering the most suitable approach to create the registry taking dementia as our case study. Specifically, the main objectives were to undertake the analysis of relevant experts’ perceptions and opinions to (1) identify the model of the registry that perfectly fits the Egyptian context and (2) provide evidence‐based policy recommendations for the registry planning structure and development.

## 2 METHODS

### 2.1 Design

The study followed a qualitative research design to explore the different aspects and dimensions of creating a national disease registry and specifically delve into the situation of dementia in Egypt as a case study. Understanding the situation in Egypt and the experiences of the leading countries is crucial prior to developing the registry. Thus, the qualitative research design was selected, as it is perfectly used to holistically study interpretations and processes,^18^ enable human interaction,^19^ and investigate human experiences to answer the hows and whys.^20^ The qualitative design also aimed to explore both the national and international contexts to identify the different constraints that the registry might have and the learned lessons that the registry can benefit from. This required interviewing key experts with a wide range of expertise to reach a comprehensive conclusion and better recommendations on how to create this first national registry in Egypt. The study also provides expert, evidence‐based policy recommendations for improved structuring of the registry and dementia in Egypt.

### 2.2 Methodology

The main research methodology was conducting expert interviews with national and international registry experts, dementia researchers, government specialists, clinical specialists, and other stakeholders to investigate the idea of creating a disease registry in Egypt, its potential impact, proposed challenges, and most appropriate structure of a registry to generate. All data collected were primary data depending on the expert's kind of knowledge and experiences based on their area of work, types of responsibilities, and their specific functions in their organizations. In this regard, we divided the areas of expertise of the people that we needed to interview into those with knowledge about: (1) existing dementia registries around the world; (2) health policy and governmental regulations; (3) clinical management of dementia patients in Egypt; (4) neuroscience research and training with a focus on aging in Egypt; (5) social research, particularily in aging and dementia; and (6) non‐governmental organizations (NGOs) that advocate for old‐age patients and the services provided for them.

### 2.3 Participants and settings

An initial list of interviewees was created based on many strategies, starting with the networks of the advisory panel, discussions with people in the field, recommendations from the interviewees themselves, as well as the literature. Selection criteria included all participants should have the knowledge and experience in one or more areas of expertise mentioned above (Table 1). Diversity in the field of expertise, the sub‐field, position, sex, and work organization were taken into consideration as much as possible. Additionally, the data collection involved triangulation, as it used multiple sampling strategies, including purposive sampling, typical case, key informant sampling, and confirming and disconfirming cases.^19^

**TABLE 1:** Interviewee characteristics.

Characteristics	*n* (%)
**Sex**	
Male	10 (41.7%)
Female	14 (58.3%)
**Region**	
National	14 (58.3%)
International	10 (41.7%)
**Field of expertise**	
Academic research	6 (25%)
Neuroscience research	2 (8.3%)
Public health research	1 (4.2%)
Aging research	1 (4.2%)
Social research	2 (8.3%)
**Clinical practice**	6 (25%)
Neurology	4 (16.7%)
Psychology	1 (4.2%)
Geriatric training	1 (4.2%)
**Health policy**	2 (8.3%)
Geriatric non‐governmental organizations	1 (4.2%)
Data consulting	1 (4.2%)
Registry development	8 (33.3%)

### 2.4 Ethical considerations

An institutional review board (IRB) approval from the American University in Cairo committee was obtained for this research. All interviews were conducted in the period between January and October 2021 after the IRB approval. An informed consent form was used to notify the participants about the purpose of the study, either written or verbally. Participation in this study was voluntary, and we obtained permission from the interviewees prior to recording the meeting. All the interviews, transcripts, and recordings are confidential, and only people with authorization can access them. The authors made all the transcription, interpretation, and analysis with no external help to ensure the confidentiality of the data.

### 2.5 Data collection

Data were collected using semi‐structured in‐depth interviews with the selected experts. In‐depth interviews are the most common qualitative method that facilitates the discussion of participants’ knowledge, opinions, and experience, which is perfectly fitting with the study objectives.^21^ The interviews were mainly by telephone or Zoom according to the interviewee's availability and place. Very few interviews were conducted face to face due to the COVID‐19 pandemic constrictions at the time of data collection.

Interview questions were designed to provide a deep idea of the expert's opinion and experience regarding developing a dementia disease registry in Egypt (Materials S1 in supporting information). The questions were semi‐structured to investigate more insights specific to each expert's case and let them speak without restrictions. The questions were categorized into main themes following a similar study conducted in Ireland regarding (1) registries and their importance, (2) the potential benefits and risks of such an initiative in Egypt, and (3) the challenges that could arise while initiating the process.^22^

### 2.6 Data analysis

All interviews were audio‐recorded or video‐recorded when on Zoom. The international interviews were conducted in English and transcribed using artificial intelligence, verbatim. The interviews with Egyptian experts were conducted in Arabic, transcribed, then translated into English. All interviews were anonymized with every effort but given the nature of expertise and the limited number of experts in the field, some experts might be identified from the context. However, we have used codes indicating the area of the interviewee's expertise instead of the normal interviewee profile and without mentioning the position or organization for better anonymization. The transcripts were then analyzed using the inductive thematic analysis approach as shown previously.^23^, ^24^, ^25^ We first divided the transcripts into several sections based on the similarity of questions, then used open coding, and derived and grouped the themes from the text. After creating the thematic index, cross‐checks were obtained to guarantee the validity and understandability of the codes and themes.

## 3 RESULTS

### 3.1 Participants characteristics

Out of 39 selected interviewees who were contacted either by e‐mail or phone calls and were given an explanation of the research with a request for an interview, 24 accepted the invitation to participate in the study with oral or written consent and were individually interviewed. They included 41.7% males, 58.3% females, 58.3% nationals, and 41.7% internationals. They had a wide range of expertise, including academic research (25%), clinical practice (6%), health policy (8.3%), NGOs (4.2%), data consulting (4.2%), and registry development (33.3%; Table 1). Some experts on the selected list were unavailable for formal meetings (*n* = 15) for various reasons.

### 3.2 Themes

**FIGURE 1:**
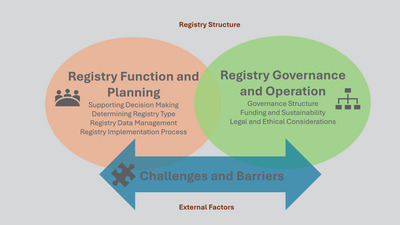
Factors affecting the development of a disease registry.

The analysis concluded with three main themes covering the registry structure and the external factors affecting its development (Figure 1). The themes are (1) registry objectives and planning, (2) registry governance and operation, and (3) challenges and barriers (Table 2).

**TABLE 2:** Summary of themes and representative quotes.

Registry objectives and planning	
Provide accurate and timely information for decision making	“So the benefit of the disease registry really can be summarized in two points: 1‐ Is that it actually facilitates research for intervention and then for sustainable solutions. 2‐ Is that actually it helps you to track the success and how do you use your limited resources in terms of funding and provide the best services.” (Neuroscience Researcher, NR2, Cairo, January 2021)
	“Doing this on a national basis, we will be able to track the numbers and the outcomes for these individuals, and it is very important in terms of getting a handle on the numbers of people that actually have the condition, looking at the healthcare utilization by these individuals, and track their outcomes over time. In terms of planning for health service provision, it is important to have the national numbers but also the resources that they require and the interventions that they need.” (Registry Specialist, RS1, International, May 2021)
Determining the registry type for Egypt	“It should be a mixed one. If I'm speaking about the Ministry of Health, they need patient care for treatment and prevention. If I am speaking about universities, then the research is much more important, so a combined one is very important.” (Government Official, HP1, Cairo, January 2021)
Registry data management	“In the beginning, you have to have a unified form for data collection … It must be very realistic and simple to be achievable … that a person can do in a short time, they have easily cut off scores, and it is not hindering for them.” (Clinical Neurologist, CN4, Cairo, Sep 2021)
	“In terms of access to the registry data … the only person that can see the full data is the principal investigator … if any of collaborators has an idea of a publication, they can suggest … then you can give them the summary data or give them the data set if you trust them.” (Registry Specialist, RS3, International, September 2021)
Registry implementation process	“Now the advantage of doing phasing is that you can modify the protocol, you can also teach the people that you're adding on, you know how to navigate the system that you set up and so forth. It also ensures that I think basically it's about understanding the system and then scaling it up gradually.” (Registry Specialist, RS3, International, September 2021)
	“Collaborate closely with government bodies that are interested, MOH [Ministry of Health], MOSS [Ministry of Social Solidarity], national planning or combination of them. You should also investigate the positivity that CAPMAS [Central Agency for Public Mobilization and Statistics] is responsible for data collection and be a partner. They are usually beneficial in data collection.” (Social Researcher, SR1, Cairo, January 2021)
Registry governance and operation	
Governance structure	“It has to start in your institution or research center, or you can partner with an NGO and give the functional or technical procedures. The government then can adopt that and take the ownership, but the initiative itself will not start unless in a research institution.” (Social Researcher, SR2, Cairo, January 2021)
	“The idea is that those who founded the registry will be the keenest on maintaining it. So, I do not think that being owned by the government would be a good idea in the beginning. They can be involved to support and do audits or something, but the project to expand has to be owned by the founders.” (Clinical Neurologist, CN1, Cairo, January 2021)
Funding and sustainability	“We need to figure out a way to actually make us almost like self‐funding in some ways, and so that's a challenge.” (Registry Specialist, RS2, International, May 2021)
	“So, we have spoken to numerous other countries … the ones that have been the most sustainable long term have been the ones that have had legislation established.” (Registry Specialist, RS4, International, October 2021)
Legal and ethical considerations	“We have to tell the person that now we have to register you and they can say no, they don't need to say yes or sign something, but they have to be informed.” (Registry Specialist, RS8, International, October 2021)
Challenges and barriers	
Disease perceptions and diagnosis	“What is difficult is the diagnosis. Dementia is under‐detected or diagnosed, you get many people in the hospital come in, say respiratory or cardiac issues, but dementia has not been diagnosed or not in the record.” (Registry Specialist, RS1, International, May 2021)
Follow‐ups and drop‐outs	“It's dropouts … the problem is that the clinics don't type in every follower. About 50% get follow‐up after one year. But the clinic doesn't enter the data … Or maybe they don't meet the people, and they don't get to the follow‐up that they should have.” (Registry Specialist, RS8 International, October 2021)
Complex regulations and limited funding	“There are a lot of legislative structures, and when you think of starting this you'll find people who say that it's a national security issue because of the security of data.” (Neuroscience Researcher, NR1, Cairo, January 2021)

#### 3.2.1 Registry objectives and planning

All experts agree that dementia registries are important tools with numerous benefits that impact different fields, including research, quality of patient care, disease knowledge, and policy making. The consensus is that they are essential in collecting information about dementia patients, which will inform the practitioners of all these fields. In addition, experts noted that the proper planning of the registry would be crucial to guarantee its success, starting from determining the goals and objectives to design the registry till its implementation.

##### 3.2.1.1 Registries provide accurate and timely information for decision making

All interviewed experts agree that dementia registries provide high‐quality or validated data that give us information about different aspects of the disease. The fact that we still do not have any accurate prevalence of dementia in Egypt and that we rely on estimations is challenging for health‐care providers and policy makers in terms of proper planning.^15^, ^26^ Moreover, all the 14 national experts agreed that there is a great need for this kind of information in Egypt to support and improve the health‐care system, especially because we lack any sort of information regarding the geriatric population. Having accurate prevalence numbers that represent all areas in Egypt is crucial to determine the disease burden and identify the need to develop specific goals, plans, and interventions that help properly allocate the resources and services required in each region while saving efforts and money by avoiding incorrect resources allocation.

Moreover, having a registry that provides national data on dementia will be a good source of information that influences policy and decision making. A registry provides evidence‐based data that assist in developing appropriate plans, strategies, and policies. Four experts discussed how the policy‐making process could benefit from having accurate national data of the patients, their distribution, and their characteristics. They also highlighted how this information could support the planning process in terms of allocation of services, budgeting, and developing the general health plan.

##### 3.2.1.2 Determining the type of registry for Egypt

Dementia registries have several types that depend on the focus or objectives of the registry, including research registries, quality of care registries, and epidemiological registries.^27^ Determining the type of registry that is most needed in Egypt is an essential step that will guide the whole planning, design, and implementation process. All the interviewed experts who discussed registry functions agreed that registries providing data on the incidence and prevalence of the disease would help initiate more research studies. Researchers should have evidence from the country that states the disease situation so that they can prioritize working on some areas and be able to predict the disease or have valid data that support their studies. Considering that will help researchers investigate the causes and determinants of the disease that led to the exacerbation of the problem in one area.

In addition, having a solid registry plays a critical role in improving the clinical care of patients and improving the quality of the services provided to them. Assessing the data of patients with dementia and tracking the outcomes of used interventions help health‐care providers identify the needs of patients and improve the services provided to them. In Egypt, the health‐care system does not depend that much on the general practitioners putting all the hectic process of identifying and diagnosing patients on the shoulders of the specialists or consultants. This, in turn, leads to the increase of non‐detection or late detection of cases. A registry is important for organizing the structure of this work and putting basic investigation measures into place that will facilitate the process of diagnosis.

Hence, all interviewed experts on this issue (20 experts) agreed that although a quality‐of‐care registry is the most needed one to benefit the patient, which is the ultimate goal of any health‐care–related initiative, a combined model that also includes data for research would be of benefit in terms of attracting and convincing the stakeholders to participate.

##### 3.2.1.3 Registry data management

Conflicting opinions emerged regarding the data sources that should be used to assist the data collection process in the early stages of developing the registry. Yet, six international experts indicated the importance of depending on the network of clinicians for collecting cases, especially in the beginning to facilitate the process of the pilot's initiation while avoiding barriers. Although a comprehensive registry that includes everything about the patients might be ideal, all experts agreed that for the registry to be established, only functional and effective data should be included. This means that only data items that provide information relevant to the disease or could benefit the association with the disease diagnosis and management should be added in the beginning. Further expansion to include more data items providing more information about the patients can then occur based on the primary outcomes of the established registry. All international experts advised to consider the well‐established international registries, look at the minimum datasets they use to compare them and be able to formulate the most appropriate setting that suits the Egyptian health‐care system and culture. Yet, it is crucial to carefully design a data collection form that includes all the required data questions to be filled in as identified from the minimum data set, and the questions that should be asked to the patient or caregivers if needed.

Moreover, all experts who discussed access to data (eight experts) stressed the importance of setting up a clear data access plan to set clear policies and regulations that control the access of registry users to patients’ data in a way that guarantees the security and privacy of data. The main idea is to provide access to requesters for only specific items that satisfy their purpose. In addition, confidentiality and privacy of patients’ data were also raised during the discussions as the major ethical concerns to protect the participating patients. Five experts highlighted the ethical regulations that control data sharing and privacy and the varied ways of protecting data privacy through only releasing aggregates of data to prevent any possible identification of a patient's case and through coding the data registered to hide all the personal information that could reveal the identity of the person. Yet, establishing proper guidelines and procedures for data management will ensure the privacy of patients, and hence, it should no longer be a concern.

##### 3.2.1.4 Registry implementation process: piloting and expansion

There is no available perfect model of dementia registries to follow, and most of the available registries were designed to satisfy their objectives.^28^ In addition, implementing a national registry is a complex and expensive process. Yet, a limited database will not be sufficient for the urgent needs of any stakeholders in the dementia field. Consequently, phasing the registry to start with a small pilot will help evaluate the process and modify the protocol accordingly. Most of the national and all international experts agreed on the importance of dividing the implementation of the registry into phases, starting with a pilot on few collaborating centers and then expanding it into a national setting.

Moreover, all experts noted that researchers and clinicians should be included in the registry development process as main stakeholders and potential end‐users. The discussion was raised on who else should be involved, especially from the high authorities. Four experts agreed on the importance of involving the Ministry of Health as one of the potential partners that should collaborate as government bodies to influence the process. The Ministry of Social Solidarity is also a potential partner, as they have the mandate of providing social care for elderly individuals. Two experts also suggested the inclusion of Central Agency for Public Mobilization and Statistics in the process, as they would be a beneficial partner when collecting population‐based data. In addition, patients and caregivers should be included in the development process to consider their input and opinions while establishing the registry plan.

#### 3.2.2 Registry governance and operation

The proper governance structure of the registry, together with an oversight plan, are significant steps that guide the high‐level engagement of the registry and ensure the attainment of the main purpose.^29^ Accordingly, we raised discussions on multiple aspects related to the registry governance, including the process of initiating the registry, the governance structure of the registry that guarantees its functionality, as well as funding and sustainability issues.

##### 3.2.2.1 Governance structure

All experts agreed that an established governance plan is necessary early in the process to ensure the development of a registry that functionally satisfies the needs. Within a governance structure, there should be a clear assignment of the overall responsibility and accountability of all stakeholders and registry personnel to fulfill the domains and competencies of the registry's functionality and operation. The majority of experts (21 out of 24 interviewed experts on this matter) stated that a board that includes stakeholders’ representatives would be the optimal structure. Yet, the registry should be housed in an academic institution or a medical authority. Although the registry structure involves many parties, the registry data and the administrative house should be placed in one institution, then the representative personnel work closely with the other partners. Hence, the adoption of the government of the registry could impose some bureaucracy into the governance structure and the whole process, which should be avoided. Instead, experts highlighted that those who developed the registry would be more enthusiastic about keeping the functionality of the registry over time. So, the consensus that emerged was that an institution should take the lead in initiating the registry, which would help the progress of the implementation process.

##### 3.2.2.2 Funding and sustainability

Experts have linked the availability and stability of funds to the sustainability of the registry. Most of the registries that proved successful and sustainable over a prolonged time were the ones that secured funding. Yet, although securing funding is challenging initially, establishing a registry could be done with minimum costs when starting with a few sites from the institution's network. However, funding will be required during the further steps of expansion.

##### 3.2.2.3 Legal and ethical considerations

All experts noted that collecting patient data will require obtaining patient consent. An opt‐in or opt‐out consent is essential to guarantee voluntary patient participation and ensure patients’ ethical regulations and privacy. Introducing patients to the purpose of the study and informing them that their data will be anonymous is a way to help convince them to participate. In addition, experts mentioned the role of caregivers in signing the consent form in the case of severely demented patients, which indicates that the process of obtaining consent is essential. However, three of the international experts highlighted that the availability of a national or legal mandate to collect data for the benefit of the health‐care system is very beneficial, as the registries that follow these jurisdictions are not required to get consent for data collection.

#### 3.2.3 Challenges and barriers

##### 3.2.3.1 Disease perception and diagnosis

All the experts discussed stigma as the main obstacle for dementia management as well as developing such a registry. The negative perceptions of people toward the disease and the lack of awareness makes it difficult to accept sharing data and information about the diagnosis. Hence, most of the patients only seek help when their cases are extremely deteriorated or in severe stages, which decreases the effectiveness of management plans.

##### 3.2.3.2 Follow‐ups and drop‐outs

Longitudinal follow‐ups and drop‐out of patients are also a challenge. Including follow‐up data in the registry is essential to enable the tracking of cases and evaluation of their outcomes. However, the 10 international experts indicated that following up with patients regularly on a yearly or 6‐month basis brings about many drop‐outs. The experts clarified that the drop out is either due to the death of patients, especially as most of them have deteriorated conditions; the patients did not visit the clinic for follow‐ups; or because the clinic did not enter the follow‐up data even though the patient visited them.

##### 3.2.3.3 Complex regulations and limited funding

Three experts expressed their concerns regarding the complex legislative structure in Egypt, which requires many security permissions prior to collecting national data. In some cases, collecting these kinds of data are considered a national security issue, as people fear releasing national data regarding population health. In other cases, the regulations required to get field permits to collect data are complicated, hindering the process.

## 4 DISCUSSION

The need for real‐world data in clinical practice, patient outcomes, and health‐care services effectiveness led to the emergence of disease registries as important tools for getting valuable insights into the disease.^30^ The establishment of a national dementia registry in Egypt is of paramount importance especially to address the rising challenges related to aging and dementia care. Experts emphasized the urgent need for comprehensive national data that reflect the medical, social, and financial aspects of elderly patients to support disease health‐care planning.^31^, ^32^ The current study provides the first model for developing a registry for dementia in Egypt that will provide a model for registries of other neurodegenerative diseases and both communicable and non‐communicable diseases in Egypt and other LMICs.

The specialist's perspective underscores how registry data can inform policy makers about patient demographics and health‐care needs, aiding in decision‐making processes. This aligns with the recognized benefits of disease registries.^27^ Insights gathered from experts indicated that disease registries can be expensive and require the involvement of many stakeholders due to ethical and logistic concerns.^33^ In addition, planning a registry is an essential step that helps define a clear registry structure, guidelines, and roadmap to follow during the implementation process.^29^ Thus, carefully planning the registry that obtains specific objectives that satisfy a country's needs is essential to guarantee its success and sustainability.^29^ This planning includes defining a clear structure, guidelines, and a roadmap for implementation. Involving stakeholders in the planning process is essential, as their input can help in the registry design. Identifying data sources and determining the type of data to be included alongside starting with a small pilot are all important steps to guarantee the registry's success. Having an initial pilot phase provides valuable insights into the process’ functionality allowing better judgments and opportunities for modifications, and aligning with previously established registries.^27^, ^34^

Setting the procedures that ensure the quality and integrity of data is a critical step during registry planning to guarantee the effectiveness of the registry in supporting patient quality of care as well as health policy making.^35^

The experts elaborated on the benefits of starting the initiative from a research or academic institution as that would support the easier implementation and data collection processes, especially in the beginning to avoid any bureaucratic activities inferred by the government. Starting with the data available in one institution and then adding other sites from the personal network would help initiate a pilot that would be connected and expanded for further coverage. However, involving the government officials even in an informal setting would be helpful to assist in the identification of needs and policy implications. This is in line with the previously published plan conducted for the Irish dementia registry.^22^ Furthermore, involving NGOs in the initiation process could be useful in terms of providing access and funds. Nevertheless, after the initiation of the first step, the government should be involved in a formal context to enable the national expansion of the registry.

Yet, developing a national registry faces many challenges in disease perception, data collection, and regulatory complexities. Stigma associated with dementia as well as the involvement of multiple medical specialties that the patients visit, often leads to delayed diagnosis, making it more challenging to identify and recruit patients. In addition, communication remains a hurdle, as careful encouragement and training are required for participating patients and health‐care providers. Logistical issues like manual documentation, high drop‐out rates for follow‐ups, and complex legislative requirements further hinder progress. Experts underscore that pilot phases, clear objectives, multi‐stakeholder collaboration, and sustainable funding are critical for success, as is involving health‐care providers, decision makers, and NGOs to build support for the registry.

Globally, dementia registries in high‐income countries provide frameworks for addressing key health issues, either to improve dementia quality of care as in the case of the Swedish Dementia registry (SveDem),^36^ or to ensure registry effectiveness through comprehensive data collection and stakeholder engagement as in the Australian Dementia Network (ADNeT).^37^ Adopting the learning lessons from such models could help address challenges identified in the study as standardizing the data collection process, fostering the participation of clinical practitioners from the planning phase, and so on. In addition, aligning the registry goals with existing national health‐care policies, as seen in global models, could also enhance the feasibility and acceptance of establishing such a registry.

Additionally, considering previous comparable challenges faced by other low‐ and middle‐income countries (LMICs) and the efforts to solve them would also offer valuable insights for the registry. For instance, prior South African efforts highlighted the role of community health workers in bridging health‐care gaps to manage non‐communicable diseases.^38^ Similarly, the Nigerian efforts to establish a Parkinson's disease registry fostered the successful role of mobile health technologies for disease surveillance that could be adapted as a strategy for other registries in the region.^39^ Yet, these comparisons to LMICs underline how these countries faced challenges similar to the Egyptian context, including stigma, resource limitations, and workforce shortages.

While the Egyptian dementia registry initiative faces significant challenges, it also holds the potential to transform dementia care in Egypt. The registry offers the opportunity to inform national dementia strategies. The availability of comprehensive data could guide resource allocation, identify priorities, and support advocacy and fundraising campaigns. Additionally, by framing dementia as a public health priority, the registry could encourage the collaboration between stakeholders and avoid resistance, especially by engaging ministries, and educational and social institutions. Furthermore, addressing raised concerns about data governance through clear policies on confidentiality and security could also enhance stakeholders’ trust and participation. In summary, learning from global and regional experiences, prioritizing stakeholder engagement, and integrating policy‐level strategies could pave the way for a successful implementation of the registry.

### 4.1 Study limitations

The limitations of this study could be the difficulty of collecting data and the initial resistance of participants in specific fields, especially government officials. The interviews were limited to 20 to 30 minutes, as we interviewed experts with hectic schedules. Another main constraint was the COVID‐19 pandemic restrictions, which affected the interview procedure as we had to conduct most of the interviews via Zoom, even with the participants from Cairo. The Zoom meetings, in any case, were limiting, as some participants were not comfortable using the camera, and this made it difficult to see their reactions, which could give insights on what else should be asked. We otherwise depend only on their answers to the questions. The pandemic restrictions also made it difficult for some potential experts to participate in the study. However, the diverse selection of the list enabled us to get input from experts in all the required areas.

As a qualitative study, it relied on a small sample population, which limits generalizability. However, every effort was made to ensure that the sample was purposefully designed to include experts with the best knowledge in the field. The study, however, gives insights into the feasibility of the construction of such a registry and what our next steps should be.

There is a great need for a national registry for dementia in Egypt with accurate and comprehensive data on Egyptian patients. Given the current direction of the government agenda and the increased focus on aging problems as well as big data analysis, it would seem to be an appropriate time for initiating this kind of registry.

The development of a national registry for dementia in Egypt is feasible, yet with some complications. In addition, the numerous benefits and functions of the registry would make the investment worthwhile. Although the process of developing a registry entails many steps that require support and collaborative assessment activities, learning from the successful lessons and guidelines of the international registries would assist in the planning and strategizing of the registry.

Taken together, developing such a dataset is beneficial to the Egyptian health‐care system, which makes the investment worthwhile. Support and collaborative work from all stakeholders, along with suitable funding, are essential elements of the proper implementation of the project. Yet, it might seem to be an opportune time for initiating this kind of registry to address the needs and, at the same time, benefit from the strategic opportunities.

## CONFLICT OF INTEREST STATEMENT

All authors declare no financial or non‐financial competing interests. Author disclosures are available in the supporting information.

## Supporting information



Supporting Information

Supporting Information
