# Supplementary material for: Complexity of developing a nationwide model for a dementia registry in Egypt: A qualitative study
Source: Alzheimers Dement. 2025 Feb 26;21(2):e70011. doi: 10.1002/alz.70011 (PMC11863355; doi:10.1002/alz.70011)
Supplement: Supplementary file 2 — Supporting Information [file ALZ-21-e70011-s001.docx]

**Supplementary 1: Interview Guide**

1. **Personal experience of registries**

- What does registry mean to you? What’s the importance of a registry?
- What type of registry would be more valuable to be created in Egypt? (Research, epidemiology, quality of care, etc…)

1. **Challenges for piloting this project in Egypt**

- What are the barriers of having a registry in Egypt?
- What challenges would be faced throughout the process?

1. **Data consideration**

- From your experience, what data should be included in the registry?
- What will be the most appropriate data collection method? (Hired volunteers, clinic-based, online, etc…)

1. **Data ownership and governance**

- Who should own the data or be authorized to access the data?
- Who should collect the data for better management?

1. **Ethical considerations**

- What steps should be taken to avoid any ethical consideration or decrease patient stigmatization?
